# Analysis of induced pluripotent stem cells carrying 22q11.2 deletion

**DOI:** 10.1038/tp.2016.206

**Published:** 2016-11-01

**Authors:** M Toyoshima, W Akamatsu, Y Okada, T Ohnishi, S Balan, Y Hisano, Y Iwayama, T Toyota, T Matsumoto, N Itasaka, S Sugiyama, M Tanaka, M Yano, B Dean, H Okano, T Yoshikawa

**Affiliations:** 1 Laboratory of Molecular Psychiatry, RIKEN Brain Science Institute, Saitama, Japan; 2 Department of Physiology, Keio University School of Medicine, Tokyo, Japan; 3 Center for Genomic and Regenerative Medicine, Juntendo University School of Medicine, Tokyo, Japan; 4 Department of Neurology, School of Medicine, Aichi Medical University, Aichi, Japan; 5 Laboratory of Protein Conformation Diseases, RIKEN Brain Science Institute, Saitama, Japan; 6 Division of Neurobiology and Anatomy, Graduate School of Medical and Dental Sciences, Niigata University, Niigata, Japan; 7 The Florey Institute of Neuroscience and Mental Health, Howard Florey Laboratories, The University of Melbourne, VIC, Australia

## Abstract

Given the complexity and heterogeneity of the genomic architecture underlying schizophrenia, molecular analyses of these patients with defined and large effect-size genomic defects could provide valuable clues. We established human-induced pluripotent stem cells from two schizophrenia patients with the 22q11.2 deletion (two cell lines from each subject, total of four cell lines) and three controls (total of four cell lines). Neurosphere size, neural differentiation efficiency, neurite outgrowth, cellular migration and the neurogenic-to-gliogenic competence ratio were significantly reduced in patient-derived cells. As an underlying mechanism, we focused on the role of *DGCR8*, a key gene for microRNA (miRNA) processing and mapped in the deleted region. In mice, *Dgcr8* hetero-knockout is known to show a similar phenotype of reduced neurosphere size (Ouchi *et al.*, 2013). The miRNA profiling detected reduced expression levels of miRNAs belonging to miR-17/92 cluster and miR-106a/b in the patient-derived neurospheres. Those miRNAs are reported to target p38α, and conformingly the levels of p38α were upregulated in the patient-derived cells. p38α is known to drive gliogenic differentiation. The inhibition of p38 activity by SB203580 in patient-derived neurospheres partially restored neurogenic competence. Furthermore, we detected elevated expression of *GFAP*, a gliogenic (astrocyte) marker, in postmortem brains from schizophrenia patients without the 22q11.2 deletion, whereas inflammation markers (*IL1B* and *IL6*) remained unchanged. In contrast, a neuronal marker, *MAP2* expressions were decreased in schizophrenia brains. These results suggest that a dysregulated balance of neurogenic-to-gliogenic competence may underlie neurodevelopmental disorders such as schizophrenia.

## Introduction

Schizophrenia is a debilitating mental illness, with a prevalence of approximately 1% worldwide, making it a relatively common disease. Although a complete understanding of the pathological architecture is still elusive, the genetic components are thought to consist of numerous weak, risk-conferring genomic variations and/or rare and large effect-size variations. Rare structural variations (copy number variants) contribute to genetic risk for neurodevelopmental disorders such as schizophrenia.^1, 2^

The 22q11.2 deletion syndrome is caused by a hemizygous microdeletion in the 22q11.2 chromosome region and has an incidence of 1 in 2000–4000 live births.^3, 4^ Although atypical microdeletions have been described, most of the deletions (around 90%) are 3 Mb in size, which includes approximately 60 genes. The remaining microdeletions (~10%) are 1.5 Mb in size and include approximately 35 genes. Most the genes within the microdeletions are expressed in the brain.^4, 5^ Patients with the microdeletion exhibit a spectrum of cognitive deficits, and approximately 30% of them develop typical schizophrenia in adolescence or early adulthood.^6, 7^ It is suggested that the 1.5 Mb microdeletion is sufficient for psychiatric phenotypes, possibly because this deletion encompasses multiple genes involved in the development, maturation and function of neurons and neuronal circuits.^4, 8^

Previous studies mainly focused on five genes located in the 1.5 Mb region, which confer risk of schizophrenia based on studies of human genetics and using animal models.^4, 9, 10, 11, 12, 13^ The five genes are: *COMT* (encoding a central enzyme in monoamine catabolism), *PRODH* (encoding an enzyme required for proline metabolism), *TBX1* (encoding a transcription factor involved in the embryonic development of multiple tissues and organs), *ZDHHC8* (encoding a palmitoyl transferase enzyme) and *DGCR8* (encoding a key regulator of microRNA synthesis).

To gain a deeper understanding of the changes leading to schizophrenia during neurodevelopment,^14^ cellular models are needed to help identify biological mechanisms that are perturbed during this process. Such cellular models are now possible because of the availability of human-induced pluripotent stem cells (hiPSCs) derived from schizophrenia patients and their differentiated neural stem or progenitor cells and neurons.^15, 16, 17, 18, 19^ Using such a model, we initially examined cell morphologies and differentiation efficiencies of hiPSCs to neurospheres (mainly consisting of neural stem/progenitor cells) and further to neuronal cells. Neurospheres derived from hiPSCs from subjects with schizophrenia showed abnormal phenotypes similar to neurospheres derived from *Dgcr8* heterozygous knockout mice.^10^ Therefore, we focused on the changes in microRNA (miRNA) expression potentially elicited by *DGCR8* haploinsufficiency, and investigated subsequent molecular cascades.

## Materials and methods

### Ethical approval

This study was approved by the Ethics Committees of RIKEN and all participating institutes, including the Keio University School of Medicine Ethical Committee for Skin Biopsy and hiPS Cell Production (approval no. 20080016), and conducted according to the principles expressed in the Declaration of Helsinki. All controls and patients gave informed, written consent to participate in the study after being provided with, and receiving an explanation of study protocols and objectives.

### Establishment of hiPSC lines and hiPSC culture

Two hiPSC clones were established from each of two female schizophrenic patients: SA001-1D2, SA001-3B1 from SA001 (aged 37 years old), and KO001-19 and KO001-25 from KO001 (aged 30 years old; Supplementary Figure 1a). The clinical histories of the patients have been described in our previous reports.^20, 21^ The control hiPSC lines 201B7 and YA9 were generated from a 36-year-old Caucasian female^22, 23^ (Supplementary Figure 1a). The remaining control hiPSC lines WD39 and KA23 were generated from a 16-year-old Japanese female and a 40-year-old Japanese male,^22, 24^ respectively (Supplementary Figure 1a). The control and patient human dermal fibroblasts were reprogrammed with retrovirus expressing the transcription factors *OCT4*, *SOX2*, *KLF4* and *C-MYC*. The maintenance of human dermal fibroblasts, retroviral preparation, infection methods, hiPSC culturing and characterization of established cells were performed as described previously.^23^

### Differentiation of hiPSCs to neuronal lineage

For the induction of neurospheres, hiPSCs were incubated with TrypLE Select (Life Technologies, Carlsbad, CA, USA) for 10 min at 37 °C. The digestion was quenched with 0.02% w/v trypsin inhibitor (Sigma-Aldrich, St Louis, MO, USA) in phosphate-buffered saline (PBS). The hiPSCs were dissociated into single cells by pipetting and plated at a density of 10 000 cells ml^−1^ in an uncoated T75 flask containing the neural culture medium^20^ supplemented with human leukemia inhibitory factor (Merck Millipore, Darmstadt, Germany) and with basic fibroblast growth factor (PeproTech, Rocky Hill, NJ, USA). The cells were cultured in an atmosphere containing 4% O_2_ and 5% CO_2_ for 14 days. The neurospheres were passaged repeatedly by culture in the same manner. The neurospheres in passages four to seven were used for analysis. The neurospheres were then collected for neural differentiation. For neural differentiation, the neurospheres were dissociated into single cells by pipetting and plated at a density of 200 000 cells per well on coverslips coated with poly-l-ornithine (Sigma-Aldrich) and fibronectin (Sigma-Aldrich) into 24-well plates. To induce neuronal differentiation, the cells were further cultured for 10 days in the neural culture medium (without human leukemia inhibitory factor or basic fibroblast growth factor) supplemented with 2% (v/v) B27 (Life Technologies), 10 ng ml^−1^ brain-derived neurotrophic factor (R&D Systems, Minneapolis, MN, USA), 10 ng ml^−1^ glial-derived neurotrophic factor (R&D Systems), 200 μm ascorbic acid (Sigma-Aldrich) and 1 mm dibutyryl-cAMP (Sigma-Aldrich).

### Neurosphere formation assay

The hiPSCs were incubated with TrypLE Select for 5 min, then dissociated into single cells by pipetting. The cells were plated at a density of 10 000 cells in an uncoated 24-well plate, containing neural culture medium supplemented with human leukemia inhibitory factor and basic fibroblast growth factor. The cells were then cultured in an atmosphere containing 4% oxygen and 5% carbon dioxide, for 5 days.

### Neurite growth and cellular migration assays

The neurospheres at passages four to five were used for this analysis. Five to 10 neurospheres were allowed to adhere to coverslips coated with poly-l-ornithine and fibronectin in a 24-well plate containing neural culture medium, supplemented with 2% (v/v) B27. The cells were cultured in an atmosphere containing 4% oxygen and 37% carbon dioxide for 48 h. After culturing, the cells were stained with the neurite marker, βIII-tubulin and the neuronal nuclear marker, NeuN. Average neurite lengths and migration distances from each neurosphere were measured using NIH Image J.

### miRNA array analysis

For microarray-based miRNA analysis, we used the Human miRNA Microarray, Release 19.0, 8 × 60K array (Agilent Technologies, Santa Clara, CA, USA), capable of measuring 2042 mature human miRNAs. The miRNAs were isolated from neurospheres using the miRNeasy Mini Kit (Qiagen, Valencia, CA, USA), and labeled using the miRNA Complete Labeling Reagent and Hyb Kit (Agilent Technologies). Array hybridization was performed according to the manufacturer's instructions. The microarrays were then scanned in a High-Resolution C scanner (Agilent Technologies), and analyzed using GeneSpring GX (Agilent Technologies). The percentile shift method (90th percentile) was used to normalize the inter-microarray range of expression intensities. The *P*-values were calculated using Student's *t*-test (two-tailed) between data from patient (*n*=4) and control (*n*=4) groups.

### Messenger RNA array analysis

Total RNA from neurospheres was extracted using the RNeasy Mini Kit (Qiagen). The quality of RNA was assessed using a Bioanalyzer RNA 6000 Nano Chip (Agilent Technologies). The RIN (RNA Integrity Number; Agilent Technologies) values that reflect the integrity of RNA were greater than 8.0 in all the samples. Total RNA was reverse-transcribed, labeled with biotin and hybridized to the Human Genome U133 plus 2.0 Array (Affymetrix, Santa Clara, CA, USA). Hybridization, washing and scanning were conducted according to the manufacturer's instructions. The data analysis was performed using GeneSpring GX (Agilent Technologies). To normalize the inter-microarray range of expression intensities, the percentile shift method (90th percentile) was used. The *P*-values were calculated using Student's *t*-test (two-tailed) between data from patient (*n*=4) and control (*n*=4) groups. The gene ontology option on GeneSpring GX was utilized to determine the most significant biological processes (corrected *P*<0.05) represented in the neurosphere transcriptome.

### Real-time quantitative RT-PCR of messenger RNA, miRNA and primary miRNA

Total cellular RNAs including messenger RNA (mRNA), miRNA and primary miRNA (pri-miRNA) were extracted from neurospheres using miRNeasy Mini Kit (Qiagen), and then single-stranded complementary DNA (cDNA) was synthesized using SuperScript VILO Master Mix (Life Technologies). Real-time quantitative RT-PCR (qRT-PCR) analysis of RNAs was conducted using a QuantStudio 12 K Flex Real-Time PCR System (Applied Biosystems, Grand Island, NY, USA). TaqMan probes were TaqMan Assays products (Applied Biosystems). All qRT-PCR data were captured using the QuantStudio 12 K Flex software v1.2.2 (Applied Biosystems). The ratios of relative concentrations of target molecules to the *GAPDH* gene for mRNA and pri-miRNA, and to *U6 snRNA* (small nuclear RNA) for miRNA, were calculated. All the reactions were performed in triplicate, based on the standard curve method.

### Immunocytochemical analysis

The cells were fixed in PBS containing 4% paraformaldehyde for 10 min at room temperature. Thereafter, the cells were incubated with the blocking buffer (10% goat serum in PBS plus 0.05% Tween 20: PBS-T) for 1 h at room temperature. The primary antibodies were applied overnight at 4 °C. Detection was by the following primary polyclonal mouse antibodies; SOX2 (Abcam, Cambridge, MA, USA; dilution 1/200), NeuN (Abcam; dilution 1/500), βIII-tubulin (Merck Millipore; dilution 1/500), MAP2 (Sigma-Aldrich; dilution 1/1000), GFAP (Merck Millipore; dilution 1/750), S100B (Sigma-Aldrich; dilution 1/100), OLIG2 (Merck Millipore; dilution 1/200) and SOX10 (R&D Systems; dilution 1/100). After three washes in PBS-T, a secondary antibody (Alexa Fluor 488-labeled goat anti-mouse IgG, Life Technologies; dilution 1/1000) was applied for 1 h at room temperature. The cells were counterstained with DAPI (4′,6-diamidino-2-phenylindole) to highlight the nuclei. After washing in PBS-T, the cells were mounted in PermaFluor Aqueous Mounting Medium (Thermo Fisher Scientific, Waltham, MA, USA). Fluorescent signals were detected using a confocal laser-scanning microscope FV1000 (Olympus, Tokyo, Japan). We counted cells positive for both βIII-tubulin and DAPI signals as neurons. These signals can differentiate each cell as βIII stains neuronal cell bodies and neurites, and DAPI stains nuclei. Astrocytes were defined as both GFAP- and DAPI-positive.

### Western blot analysis

The neurospheres were isolated, suspended in RIPA lysis buffer (Cell Signaling Technology, Danvers, MA, USA) supplemented with a protease inhibitor cocktail (Sigma-Aldrich), triturated and centrifuged at 10 000 *g* for 10 min at 4 °C. The resultant supernatants were separated on 10% SDS polyacrylamide gel electrophoresis gels (10 μg protein per lane), and the proteins were blotted onto a polyvinylidene difluoride membrane. After blocking with 5% skimmed milk, the membranes were incubated with either anti-p38α antibody (Cell Signaling Technology; dilution 1/1000) or anti-GAPDH antibody (Santa Cruz Biotechnology, Santa Cruz, CA, USA; dilution 1/1000) at 4 °C overnight, followed by incubation with HRP (horse radish peroxidase)-conjugated anti-rabbit IgG (GE Healthcare, Little Chalfont, UK; dilution 1/5000) or HRP-conjugated anti-goat IgG (GE Healthcare; dilution 1/5000) at room temperature, for 1 h. Detection of the signals was carried out with an Immobilon Western Chemiluminescent HRP Substrate (Merck Millipore), and the bands were analyzed using the LAS-1000 plus image analyzer (Fuji Film, Tokyo, Japan). The intensities of the bands were quantified using the Image Gauge software (Fuji Film). The expression levels of p38 were normalized to that of GAPDH.

### Postmortem brain analysis

For human postmortem studies, the tissue was obtained from Brodmann's area 8 in the left hemisphere from 95 subjects with schizophrenia and 93 age- and sex-matched controls. The tissue was obtained from the Victorian Brain Bank Network (The Florey Institute for Neuroscience and Mental Health, Parkville, VIC, Australia, http://www.florey.edu.au/research/brain-bank-network). The tissue was collected after obtaining permission from the Ethics Committee of the Victorian Institute of Forensic Medicine. Total RNA was extracted using the miRNAeasy Mini kit (Qiagen), and we used the samples whose RIN was ⩾7.0. The demographic data of those 51 samples from schizophrenia and 70 from controls are described in Supplementary Table S1. Single-stranded cDNA was synthesized using SuperScript VILO Master Mix (Invitrogen). Quantitative RT-PCR analysis was conducted as stated above. TaqMan probes and primers for *MAPK14*, *GFAP*, *MAP2*, *IL1B, IL6* and *GAPDH* (internal control; see ref. 25) were chosen from TaqMan Gene Expression Assays (Life Technologies). All real-time quantitative RT-PCR reactions were performed in triplicate, based on the standard curve method. The expression ratio of *GFAP*/*MAP2* in each postmortem brain sample was calculated as (*GFAP*/*GAPDH*)/(*MAP2*/*GAPDH*).

### Genomic quantitative PCR analysis of 22q11.2 deletions in postmortem brain samples

Insertions/deletions within the genomic length of Hs04090007_cn (Chr.22:19,318,224-19,419,219) were analyzed by real-time genomic quantitative PCR using the TaqMan Copy Number Assay (Applied Biosystems). For the analysis of copy number variant in the 22q11.2 region, the RNaseP gene was used as a normal copy number control gene. No copy number polymorphisms have been documented in RNaseP genes. For genomic quantitative PCR, DNA solutions were first measured using an ultraviolet spectrophotometer and further quantified using a TaqMan RNase P Detection Reagent kit (Applied Biosystems).

### Statistical analysis

Statistical evaluation was performed either by Student's *t*-test (two-tailed) or by Mann–Whitney *U*-test (two-tailed) for means between patient and control groups, or by Pearson's method for correlation, using SPSS software version 19 (IBM, Tokyo, Japan). Within each group, the subjects with transcript levels >2 s.d. from the group mean were considered as outliers and removed. In addition, for the expression analyses of *GFAP*, *MAP2* and ratio of *GFAP*/*MAP2*, the *P*-values were also calculated using analysis of covariance by adjusting the age.

## Results

### Generation of hiPSCs derived from schizophrenia patients with the 22q11.2 microdeletion

We generated hiPSCs from schizophrenia patients with the 22q11.2 microdeletion (four lines from two patients), using the fibroblasts according to standard methods^20, 21, 23^ (Supplementary Figure 1a). CGH array analyses revealed that both patients carried a 2.6 Mb-hemizygous deletion at chromosome 22q11.2.^21^ This should correspond to a typical 3 Mb deletion because the CGH array lacks the probes for detecting regions near the ends of 3 Mb region.^26^ No other psychiatric disorder-relevant copy number variants^1^ were found in the two patients (Supplementary Tables 2 and 3). All of the hiPSC lines could be differentiated into neurons through a stage of neurosphere formation (Supplementary Figure 1b). In our protocol, all the cells in neurospheres expressed the neural marker Nestin, suggesting that our neurospheres consisted almost entirely of neural stem or progenitor cells.^20, 21, 27^ Thus, all the hiPSC lines were suitable for neuronal analyses.

### Neurosphere formation from hiPSCs

**Figure 1:**
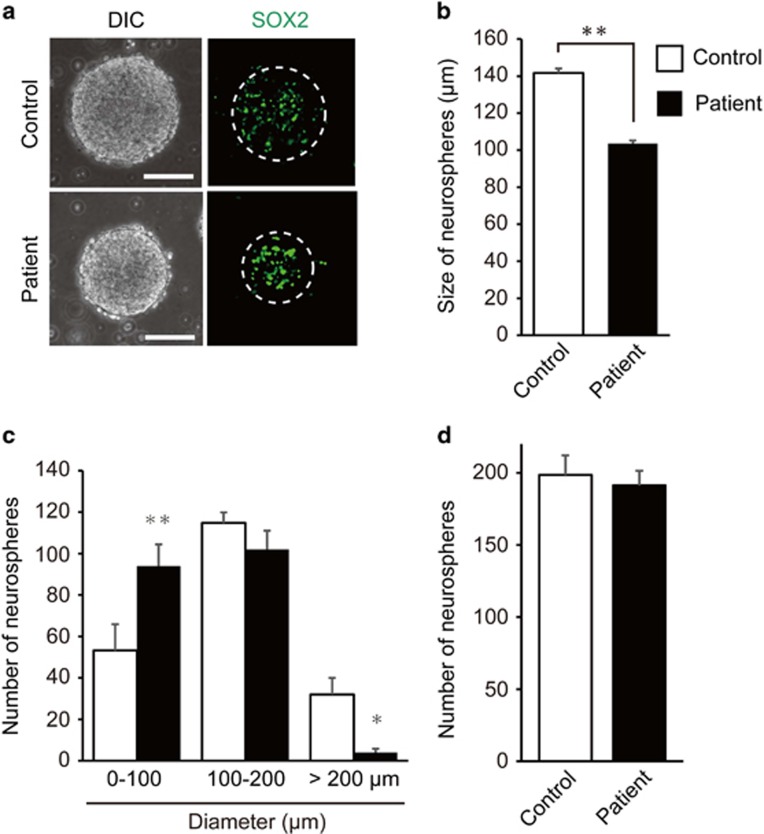
Reduction in size of patient-derived neurospheres. (**a**) Bright-field and immunofluorescent images of neurospheres stained for SOX2 (green), derived from control and patient-derived human-induced pluripotent stem cells (hiPSCs). Dotted white circles in the right panel show the outline of neurospheres. Scale bars, 100 μm. (**b**) Quantitative analysis of the mean size of neurospheres derived from control or patient hiPSCs. The mean size of neurospheres derived from patient hiPSCs was significantly smaller than that from control hiPSCs (*n*=180–210 neurospheres per cell line). (**c** and **d**) Quantitative analysis of the number of neurospheres derived from control or patient-derived hiPSCs. The number of neurospheres with a diameter of less than 100 μm or more than 200 μm were significantly different in patient-derived neurospheres, but the total number of neurospheres was not significantly different (*n*=4 for each group). Error bars show mean±s.e.m. (*P<0.05, **P<0.01; two-tailed *t-*test).

Previous reports of hiPSCs and differentiated neurons, which were derived from patients with schizophrenia, demonstrated several abnormal phenotypes *in vitro*.^18, 20, 28, 29, 30, 31^ We performed neurosphere formation assays using control- and patient-derived hiPSCs. The size and number of neurospheres were measured on the 14th day after passage (Figure 1a). The neurospheres expressed the neural stem cell marker SOX2 (Figure 1a). The mean size of neurospheres generated from patient-derived hiPSCs was reduced by 30% compared with that of control hiPSCs (*P*<0.0001; Figures 1a and b). We also found that the number of spheres with a diameter of less than 100 μm was significantly increased among patient-derived neurospheres (*P*=0.0046), whereas the number of neurospheres with a diameter of more than 200 μm was significantly decreased among patient-derived neurospheres (*P*=0.0265; Figure 1c). However, the total numbers of neurospheres generated from control hiPSCs and patient-derived hiPSCs did not differ significantly (Figure 1d). Importantly, the reduction in the size of neurospheres is also reported for neurospheres generated from the hippocampus of *Dgcr8* heterozygous knockout (*Dgcr8*^+/−^) mice.^10^

### Neuronal differentiation from hiPSC-derived neurospheres

**Figure 2:**
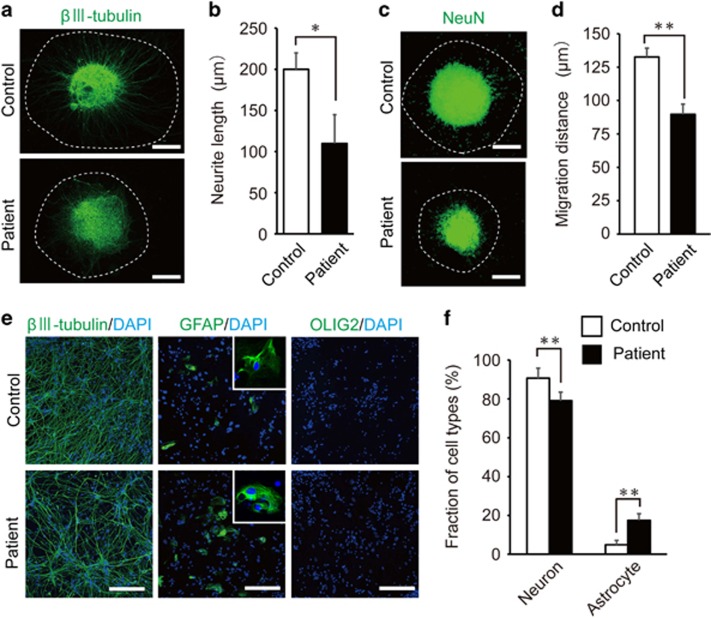
Patient-derived neurospheres undergo abnormal neural differentiation. (**a**) Representative images of neurite outgrowth from neurospheres. The neurites were visualized by immunocytochemical staining of βIII-tubulin. The average distance between the sphere and the neurite tip (white dashed line) was calculated. Scale bars, 100 μm. (**b**) Quantitative analysis of the neurite length. The neurite lengths were significantly decreased in patient-derived neurospheres (*n*=300 neurites per cell line). (**c**) Representative images of cellular migration from neurospheres. Nuclei were visualized by immunocytochemical staining with NeuN. The average distance between the sphere and the most distant cell (white dashed line) was calculated. Scale bars, 100 μm. (**d**) Quantitative analysis of cellular migration. The cellular migrations were significantly decreased in patient-derived neurospheres (*n*=300 cells per cell line). (**e**) Representative images of neural differentiation from neurospheres. Neural cells derived from neurospheres expressed βIII-tubulin and glial fibrillary acidic protein (GFAP) in the patient and control-derived samples, but not OLIG2. The magnified pictures of GFAP-positive cells are shown in the insets. Scale bars, 100 μm. (**f**) The analysis of neural differentiation efficiencies between control and patient-derived neurospheres (*n*=10 per cell line). The error bars show mean±s.e.m. (*P<0.05, **P<0.01; two-tailed *t*-test).

We investigated whether neuronal cells differentiated from patient-derived hiPSC show abnormal cell phenotypes. First, we induced neural differentiation, by using non-adherent floating culture and then plated the cells on a poly-l-ornithine- and fibronectin-coated surface. We then compared the lengths of outgrown neurites and distance of cellular migration between control and patient-derived neurospheres. The neurites were visualized by immunocytochemical staining using the neuronal marker βIII-tubulin (Figure 2a). The neurite lengths were significantly shortened in patients' neurospheres (112±36 μm), compared with controls (204±19 μm; *P*=0.0345; Figure 2b). The nuclei were visualized by immunocytochemical staining using the neuronal marker, NeuN (Figure 2c). The migration distances were significantly decreased in neurospheres from patients (90±8 μm), compared with controls (133±7 μm; *P*=0.0032; Figure 2d). Therefore, the shortened neurite lengths in patients' samples cannot solely be explained by impaired cell migration.

We next examined neural differentiation efficiencies from neurospheres into neurons and glial cells. The neurospheres were plated on fibronectin- and poly-l-ornithine-coated plates, and they were under differentiation conditions using neural culture medium containing four factors (brain-derived neurotrophic factor, glial-derived neurotrophic factor, ascorbic acid and dibutyryl-cAMP) for 10 days. The differentiated cell types were identified by immunocytochemical analysis using the neuronal markers, βIII-tubulin and MAP2 (microtubule-associated protein 2), the astrocyte markers, GFAP (glial fibrillary acidic protein) and S100B, and the oligodendrocyte markers, OLIG2 and SOX10. After 10 days, the differentiated cells expressed the neuronal marker and astrocyte marker in both the sample groups, but they did not express oligodendrocyte marker (Figure 2e and Supplementary Figure 2). These results indicate that the control and patient-derived neurospheres could differentiate into neurons and astrocytes, but not oligodendrocytes. We compared the efficiencies of neural and glial inductions between control and patient-derived neurospheres. The fraction of neurons in the total differentiated cells was significantly reduced by approximately 10% in patient-derived neurospheres (*P*=0.0086) when compared with that in control ones (Figure 2f). In contrast, the fraction of astrocytes in the total cells was significantly increased by approximately 12% in patient-derived neurospheres (*P*=0.0056; Figure 2f).

### Expression analysis of miRNA in hiPSC-derived neurospheres

The neurospheres derived from hiPSCs from subjects with schizophrenia with 22q11.2 deletion showed abnormal phenotypes similar to the neurospheres derived from *Dgcr8* heterozygous knockout mice.^10^ Therefore, we focused on *DGCR8* in our subsequent analyses. The *DGCR8* gene encodes a double-stranded RNA binding protein, an important component of the ‘microprocessor' complex that processes primary forms (pri-forms) to pre-miRNAs that have a hairpin structure, which is further processed by the Dicer complex to produce mature miRNAs.^32^

**Figure 3:**
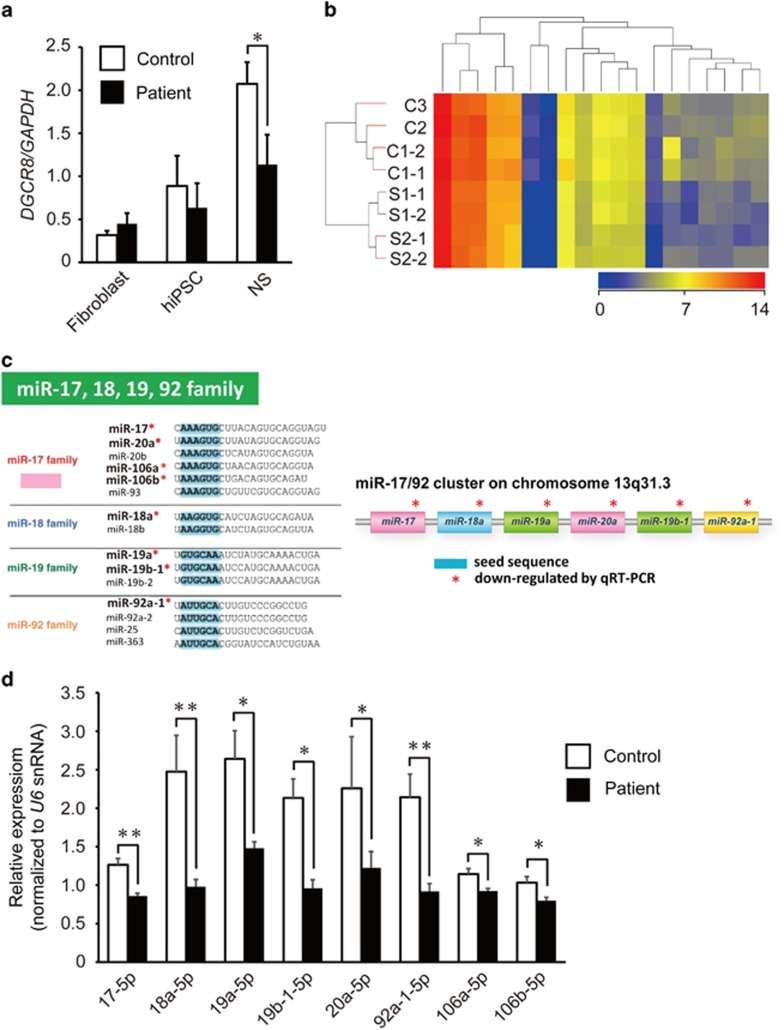
Downregulation of the miRNAs of miR-17 family and miR-17/92 cluster in patient-derived neurospheres. (**a**) Quantitative real-time PCR (RT-PCR) analysis of *DGCR8* in fibroblasts, human-induced pluripotent stem cells (hiPSCs) and neurospheres (*n*=3–4 for each group). (**b**) Heat map showing differential expression of 19 miRNAs between patient and control-derived neurospheres. (**c**) Sequences of the members of the miR-17, 18, 19 and 92 family. The sequences are divided into four families according to the miRNA seed sequences (marked in blue). Members of the miR-17/92 cluster are shown in right panel with information on their chromosomal location. Red: members of the miR-17 family; blue: members of the miR-18 family; green: members of the miR-19 family; yellow: members of the miR-92 family. (**d**) Quantitative RT-PCR analysis of eight miRNAs in neurospheres. *U6* snRNA was used as an internal control (*n*=4 for each group). Error bars show mean±s.e.m. (*P<0.05, **P<0.01; two-tailed *t-*test). miRNA, microRNA; snRNA, small nuclear RNA.

First, we confirmed that the expression levels of *DGCR8* in patient-derived neurospheres were reduced compared with those from controls (*P*=0.0423) by real-time quantitative RT-PCR (Figure 3a), although there was not a large difference of *DGCR8* expression between control and patient fibroblasts or hiPSCs (Figure 3a). Next, we performed microarray-based miRNA analysis and measured the expression levels of 2042 human mature miRNAs using the miRBase Rel. 19.0 platform in control and patient-derived neurospheres. We found that 19 miRNAs satisfied our differential expression criteria of an absolute fold change (FC) <0.83 or >1.2 and *P*<0.05 (Figure 3b and Supplementary Figures 3a and 3b).

miRNAs are categorized according to base sequences and their genomic loci. A microRNA ‘family' is a group of miRNAs that have an identical seed sequence, and a miRNA ‘cluster' is a group of miRNAs that reside in the same genomic locus. In previous studies, the miRNAs of miR-17 family and miR-17/92 cluster have been reported to show abnormal expression levels in schizophrenic brains.^33^ The miR-17 family (Figure 3c) includes miR-17 (in the current study, *hsa-miR-17-3p* showed FC=0.7 and *P*=0.0449; Supplementary Figures 3a and 3b), miR-20a/b, miR-93 and miR-106a/b.^34, 35^ The miR-17/92 cluster (Figure 3c) includes miR-17, miR-18a, miR-19a, miR-19b-1, miR-20a and miR-92a-1. Therefore, we set out to precisely quantify the expression levels of those eight miRNAs (miR-17, miR-18a, miR-19a, miR-19b-1, miR-20a, miR-92a-1 and miR-106a/b), all of which belong to the miR-17 family or the miR-17/92 cluster, using real-time quantitative RT-PCR with *U6* snRNA as an internal control probe. All the eight miRNAs showed significant downregulation in patient-derived neurospheres (Figure 3d). Importantly, downregulation of *miR-106b* detected in patient neurospheres is also reported in schizophrenic brains^36^ and Df(16)A^+/−^ mice.^9^ Complementing these results, pri-miR-106b, the pri-form of miR-106b, was upregulated in patient-derived neurospheres (*P*=0.0466; Supplementary Figure 3c), suggesting that the processing efficiency of pri-form to pre-form of miR-106b was lowered, probably due to reduced *DGCR8* expression.

### Implication of p38 in modulating neuronal differentiation competencies

**Figure 4:**
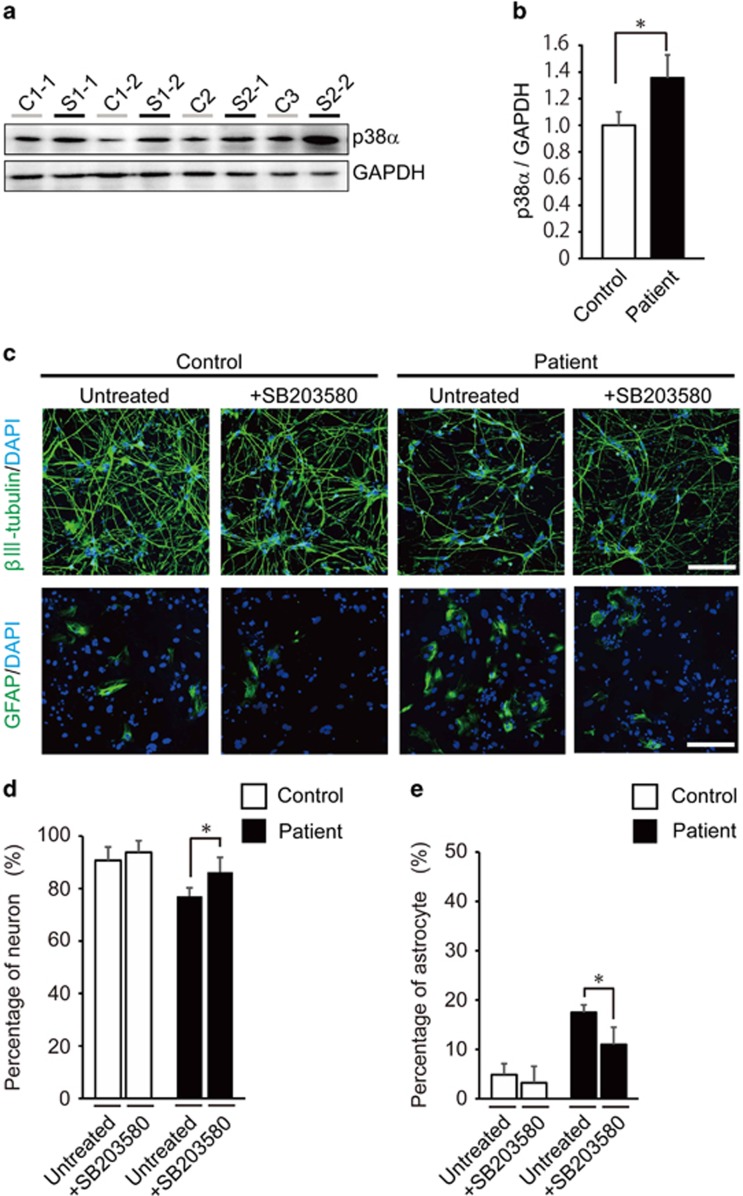
Effects of p38 protein on controlling neurogenic competence in patient-derived neurospheres. (**a**) Expression levels of p38α in human-induced pluripotent stem cell (hiPSC)-derived neurospheres examined by western blotting using an anti-p38α antibody. (**b**) Quantitative analysis of p38α protein levels in neurospheres derived from control or patient hiPSCs. The p38α protein levels were significantly increased in patient-derived neurospheres (*n*=4 for each group). (**c**) Representative images of neural differentiation from neurospheres treated with SB203580 (1.0 μm). Neurons and astrocytes were visualized by immunocytochemical staining of βIII-tubulin and glial fibrillary acidic protein (GFAP), respectively. Scale bars, 100 μm. (**d** and **e**) Analysis of the effects of p38 on neural differentiation efficiencies between control and patient-derived neurospheres. In the total differentiated cells derived from patient neurospheres treated with SB203580 (1.0 μm), neuronal population was significantly increased and astrocyte population was significantly reduced (*n*=10 per cell line). The cells used here were prepared separately from those in Figure 2f. Error bars show mean±s.e.m. (*P<0.05; two-tailed *t*-test).

It has been reported that (i) miR-17/106 targets the *MAPK14* transcript (encoding the α-isoform of p38 protein kinase) in mice and (ii) the miR-17/106-p38 axis is a critical regulator of the neurogenic-to-gliogenic transition competence.^37^ Therefore, the protein expression levels of p38α in patient-derived neurospheres were predicted to be increased by the downregulation of miR-17/92 cluster members. Conformingly, western blot analysis showed that the expression levels of p38α were significantly increased in patient-derived neurospheres (1.36-fold higher; *P*=0.0162) than those in controls (Figures 4a and b). On the basis of previously published studies,^37, 38^ we hypothesized that the upregulation of p38α is causally linked to the abnormal neuronal differentiation pattern (preferential gliogenic competence) in patient-derived neurospheres. If this is true, the abnormal differentiation phenotype could be at least partially rescued by inhibiting p38 activity. Consistent with this idea, treatments with a p38-specific inhibitor, SB203580,^39, 40^ produced a significantly higher number of neurons (*P*=0.0426) (Figures 4c and d) and a significant reduction of the astrocyte population (*P*=0.0394; Figures 4c and e) in samples derived from patients. The SB203580 treatment did not alter the fractions of neurons and astrocytes in control samples (Figures 4c–e).

### Transcriptome analysis in hiPSC-derived neurospheres

Microarray-based mRNA expression analysis in patient and control-derived neurospheres was conducted to understand the transcriptome profile relevant to schizophrenia with a 22q11.2 deletion. Of genes that are mapped in the deleted region of 22q11.2, expression levels of 22 genes including *DGCR8* were significantly decreased in patient-derived neurospheres (Supplementary Table 4). The genome-wide results depicted 263 upregulated and 123 downregulated genes with a 2-fold cutoff value and *P*<0.05 (Supplementary Figure 4). The differentially expressed genes were mainly enriched for gene ontology terms relevant for cell differentiation, neuronal development and microRNA processing (Supplementary Table 5). Further analysis revealed that upregulated genes were significantly enriched for MAPK (mitogen-activated protein kinase)-mediated nuclear events, neurotransmitter receptor binding, transmission across chemical synapses, nerve growth factor signaling and NMDA receptor activation (Supplementary Table 6). The downregulated genes fell into classes associated mainly with cell cycle-related events (Supplementary Table 6).

### Expression analysis in postmortem brains

**Figure 5:**
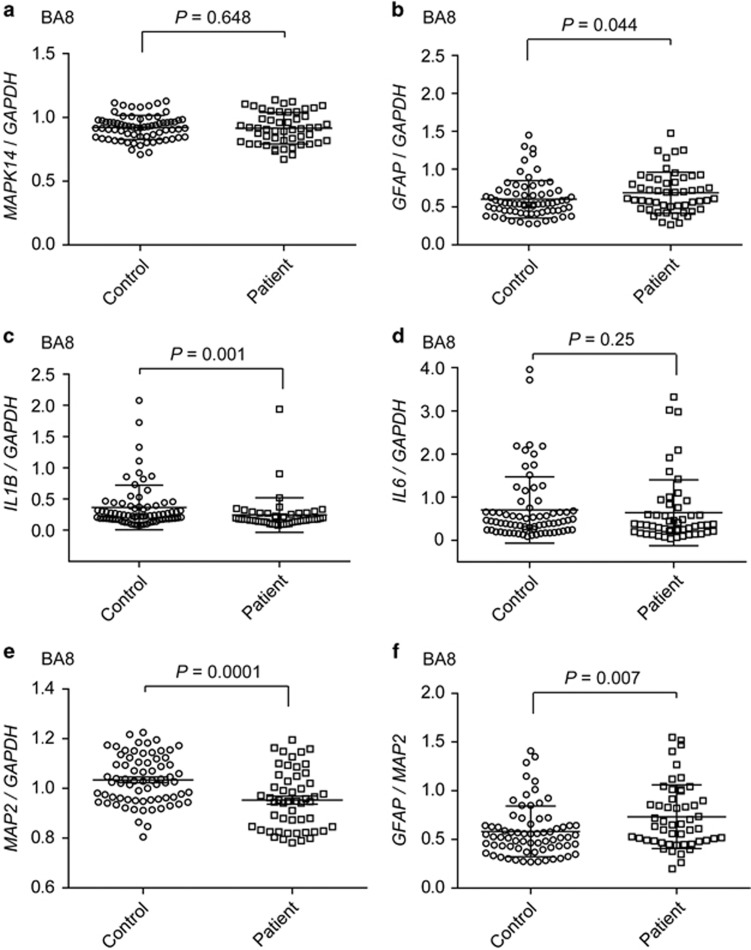
mRNA expression analyses of *MAPK14, GFAP, IL1B* and *IL6* and *MAP2* in postmortem brains. Expression levels of *MAPK14* (**a**), *GFAP* (**b**), *IL1B* (**c**), *IL6* (**d**), *MAP2* (**e**) and *GFAP*/*MAP2* (**f**) in postmortem brain tissues (Brodmann's area 8; BA8) of schizophrenia patients and controls were analyzed using real-time quantitative RT-PCR. The *P*-values were calculated using two-tailed Mann–Whitney *U*-test. Horizontal bars show mean±s.d. GFAP, glial fibrillary acidic protein; IL, interleukin; MAP, microtubule-associated protein; MAPK, mitogen-activated protein kinase; mRNA, messenger RNA.

Last, we investigated the mRNA expression levels of *MAPK14, GFAP* and the inflammation markers, *IL1B* and *IL6* in postmortem brain tissues from schizophrenia patients. In the frontal cortex (Brodmann's area 8), there were no significant differences in the expression levels of *MAPK14* between control and schizophrenia samples (*P*=0.648; Figure 5a). However, the *GFAP* expression level was nominally significantly increased in the patient samples (*P*=0.044; age-adjusted *P*=0.05; Figure 5b). In contrast, *IL1B* expression levels were significantly decreased in patient samples (*P*=0.001; Figure 5c). The expression levels of *IL6* were unchanged between control and schizophrenia samples (*P*=0.25; Figure 5d). In addition, *MAP2* expression levels were significantly decreased in the patient samples (*P*=0.0001; age-adjusted *P*=0.000015; Figure 5e). Furthermore, the expression ratios of *GFAP*/*MAP2* were significantly increased in the patient samples (*P*=0.007; age-adjusted *P*=0.002; Figure 5f). There were no significant correlations between expression levels of *GFAP* and levels of *IL1B* or *IL6* (Supplementary Figures 5a and b). The expression levels of the two inflammation marker genes showed significant correlation (Supplementary Figure 5c). The RIN and pH of schizophrenia samples were significantly and marginally lower than those of controls, respectively (Supplementary Table 1). However, the RIN (pH) and *GFAP* expression levels in the postmortem brain samples were not significantly correlated to each other: *P*=0.706 (0.987), 0.585 (0.919) and 0.120 (0.682) for control, schizophrenia and total brains, respectively. Therefore, the results suggest an elevated gliogenic competence in schizophrenia without 22q11.2 deletion.

## Discussion

The genetic underpinnings of schizophrenia are multifactorial,^41^ making it challenging to identify convergent points of common pathways, which is essential for developing novel therapeutic and preventive measures. The cells from schizophrenia patients with the 22q11.2 deletion can serve as an entry point for teasing apart this complexity and resolving the principal pathways shared among ‘general' schizophrenia, because this copy number variant is one of the largest effect-size predisposing factors. In this study, we revealed several characteristic features of hiPSCs and their differentiated cell lineage derived from schizophrenia patients with the 22q11.2 microdeletion.

We observed that patient-derived neurospheres are smaller in size and that differentiated neurons display abnormal morphology, phenotypes shared with those seen in model mice for the 22q11.2 microdeletion (Df(16)A^+/–^ mice and Df1 mice).^4, 9, 42, 43^ As a potentially relevant gene, we focused on *DGCR8* among genes that are mapped on the deleted interval, because neurospheres generated from the hippocampus of *Dgcr8*^+/−^ mice also showed size reduction.^10^ In addition, the *Dgcr8*^+/−^ mice displayed working memory deficits and sensory information-processing deficits,^9, 42^ which are seen in schizophrenia patients. DGCR8 is essential for miRNA biogenesis.^32^ As the downstream targets of DGCR8, which may be causally linked to the observed phenotypes, we examined the miR-17/92 cluster and miR-106a/b.^37^ The patient-derived neurospheres showed reduced expression levels of miR-17/92 cluster members and miR-106a/b. Downregulation of the *DGCR8* gene suppresses the conversion of a subset of pri-forms of miRNA to pre-miRNAs and results in the dampened generation of a particular subset of mature miRNAs.^44^ In the patient-derived neurospheres, the expression level of the pri-form of miR-106b was significantly increased, conforming to haploinsufficiency of *DGCR8*.

The miR-17/92 have a general role in cell proliferation and survival during normal development and also during tumorigenesis.^34, 45^ A recent study of the miR-17/92 cluster and miR-106a/b has shown that miR-19 and miR-92a repress PTEN and TBR2, and suppress the transition from radial glial cells to intermediate progenitors,^46^ and that miR-17 and 106a/b repress p38α (*MAPK14*), leading to increased neurogenic and suppressed gliogenic competences in mice.^37^ The upregulation of p38α (*MAPK14*) protein seen in the current human study may be owing to the decreased expression of miR-19a/b, members of the miR-17/92 cluster, or miR-185-5p, as they are predicted to target human *MAPK14* (TargetScan release 7.0: http://www/targetscan.org/, Supplementary Table 7; Supplementary Figure 6). These miRNAs are reported to show abnormal expression levels in schizophrenic brains.^33^ Therefore, the underexpression of miR-17/92 cluster members, miR-185-5p and miR-106a/b, and subsequent upregulation of p38α may underlie the observed size reduction of patient-derived neurospheres and the decreased neural differentiation efficiency in patient-derived neurospheres. In support of this theory, abnormal neurogenic-to-gliogenic transition competence balance in patient-derived differentiated cells could be partially recovered by the addition of p38 inhibitor. The recent studies reported that the RAF/MEK/ERK pathway also controls gliogenesis.^47^ In the patient-derived neurospheres, upregulated genes were significantly enriched for ERK-mediated nuclear events in addition to MAPK-associated pathways (Supplementary Table 6), implying the involvement of the ERK pathway in the abnormal of neurogenic-to-gliogenic transition competence balance in the patient-derived neurospheres seen in our study.

Interestingly, we detected increased expression of *GFAP*, an astrocyte marker, decreased expression of *MAP2*, a neuronal marker, and elevated ratio of *GFAP*/*MAP2*, in postmortem brains of patients with schizophrenia. This may correspond to the aberrant neurogenic-to-gliogenic transition balance seen in the neurospheres derived from schizophrenia patients with the 22q11.2 deletion. Therefore, the theory of ‘reduced neurogenic and elevated gliogenic competences' could be a hallmark of schizophrenia pathology.^48^ With respect to a potential mechanism of elevated *GFAP* expression in the postmortem brains, several studies have reported that oxidative stress and inflammatory cytokines contribute to the pathophysiology of schizophrenia.^49^ However, in our postmortem brains, the examined inflammatory markers, *IL1B* and *IL6*, were not upregulated in the disease group nor did their expression levels correlate with *GFAP* levels. Therefore, upregulated *GFAP* expression is unlikely to be related to the inflammatory status at autopsy.

The following are the limitations of the current study: (1) small iPS sample sizes; (2) iPS samples were not matched between cases and controls in terms of ethnicity and gender; (3) as no histopathological examinations were performed, it would be difficult to completely exclude the possible contribution of ‘gliosis' to elevated *GFAP* expression in schizophrenia brains; and (4) the precise mechanisms for the upregulation of *GFAP* and downregulation of *MAP2* in the postmortem brain samples from schizophrenia remain elusive. These limitations considered, replication studies using large cohorts are warranted.

In summary, by integrating information obtained from manifold analyses of reprogrammed neuronal cells, we deepened an *in vitro* mechanistic understanding of how the 22q11.2 microdeletion affects neurodevelopment. From the current results, we propose a hypothetical concept of ‘reduced neurogenic and elevated gliogenic competences' as a shared underpinning of etiologically heterogeneous schizophrenia. We also propose the potential benefit of developing compounds that have high specificity against p38α and can pass through blood–brain barrier, as novel therapeutics for schizophrenia.
