# Supplementary material for: Analysis of induced pluripotent stem cells carrying 22q11.2 deletion
Source: Transl Psychiatry. 2016 Nov 1;6(11):e934–. doi: 10.1038/tp.2016.206 (PMC5314118; doi:10.1038/tp.2016.206)
Supplement: Supplementary Information [file tp2016206x1.docx]

**Supplementary Tables**

**Table S1.** Demographic characteristics of postmortem brains

|  | *Control* | *Schizophrenia* | P*-value* |
| --- | --- | --- | --- |
| *n* | 75 | 53 |  |
| Age at death (years) | 45.5 ± 15.2 | 44.3 ± 16.4 | 0.68^a^ |
| Sex (Male / Female) | 62 / 13 | 40 / 13 | 0.38^b^ |
| Postmortem interval (hours) | 41.9 ± 14.2 | 40.6 ± 13.9 | 0.60^a^ |
| Brain tissue pH | 6.36 ± 0.20 | 6.29 ± 0.20 | 0.05^a^ |
| RNA Integrity Number | 7.69 ± 0.36 | 7.38 ± 0.26 | < 0.01^a^ |

^a^Evaluated by two-tailed *t* test

^b^Evaluated by Fisher’s exact test

**Table S2.** Copy number variations detected by CGH array in schizophrenia patients with 22q11.2 deletions (SA001)

| *Chromosome* | *Start* | *Stop* | *Size* | *Mean of log_2_(ratio)* | *Involved genes* |  |  |  |  |
| --- | --- | --- | --- | --- | --- | --- | --- | --- | --- |
| chr1 | 108,734,485 | 108,736,765 | 2,280 | 0.9348 | *SLC25A24* |  |  |  |  |
|  | 174,796,674 | 174,805,391 | 8,717 | -0.4284 | *RABGAP1L* |  |  |  |  |
| chr2 | 30,443,273 | 30,459,896 | 16,623 | 0.5957 | *LBH* |  |  |  |  |
|  | 38,221,160 | 38,231,441 | 10,281 | -0.4541 | *FAM82A1* |  |  |  |  |
| chr4 | 83,275,732 | 83,277,798 | 2,066 | 0.7564 | *HNRNPD* |  |  |  |  |
| chr5 | 180,401,334 | 180,424,411 | 23,077 | -1.7208 | *BTNL3* |  |  |  |  |
| chr6 | 37,285,479 | 37,952,326 | 666,847 | 0.5871 | *TBC1D22B* | *RNF8* | *FTSJD2* | *C6orf129* | *MDGA1* |
|  |  |  |  |  | *ZFAND3* |  |  |  |  |
|  | 124,512,162 | 124,519,435 | 7,273 | -0.4829 | *NKAIN2* |  |  |  |  |
| chr8 | 39,234,153 | 39,381,525 | 147,372 | -0.5581 | *ADAM3A* |  |  |  |  |
| chr11 | 5,784,834 | 5,809,160 | 24,326 | -0.7574 | *OR52N5* | *OR52N1* |  |  |  |
|  | 14,250,300 | 14,255,835 | 5,535 | -0.5093 | *SPON1* |  |  |  |  |
|  | 24,584,550 | 24,611,133 | 26,583 | -0.4672 | *LUZP2* |  |  |  |  |
| chr12 | 63,274,813 | 63,277,382 | 2,569 | 0.5408 | *PPM1H* |  |  |  |  |
|  | 126,444,131 | 126,450,912 | 6,781 | -0.5211 | *LOC400084* |  |  |  |  |
| chr13 | 36,398,625 | 36,403,728 | 5,103 | -0.7877 | *MIR548F5* | *DCLK1* |  |  |  |
| chr15 | 22,299,621 | 22,586,293 | 286,672 | -0.4829 | *LOC727924* | *OR4M2* | *OR4N4* | *LOC646396* |  |
|  | 54,415,916 | 54,425,471 | 9,555 | -0.8242 | *UNC13C* |  |  |  |  |
| chr16 | 82,913,701 | 82,918,737 | 5,036 | -0.5323 | *CDH13* |  |  |  |  |
| chr19 | 6,737,854 | 6,746,187 | 8,333 | 0.5051 | *TRIP10* |  |  |  |  |
|  | 14,204,171 | 14,213,718 | 9,547 | 0.5232 | *PRKACA* |  |  |  |  |
|  | 15,216,209 | 15,226,715 | 10,506 | 0.5621 | *SYDE1* | *ILVBL* |  |  |  |
|  | 30,505,856 | 30,519,597 | 13,741 | -0.448 | *C19orf2* |  |  |  |  |
|  | 41,337,409 | 41,366,639 | 29,230 | -0.4208 | *CYP2A6* |  |  |  |  |
|  | 42,395,756 | 42,440,175 | 44,419 | 0.5129 | *ARHGEF1* |  |  |  |  |
|  | 52,134,789 | 52,148,600 | 13,811 | -1.3155 | *SIGLEC14* |  |  |  |  |
| chr20 | 1,562,887 | 1,576,390 | 13,503 | 1.2245 | *SIRPB1* |  |  |  |  |
|  | 8,247,806 | 8,252,283 | 4,477 | -0.4064 | *PLCB1* |  |  |  |  |
|  | 15,630,060 | 15,634,254 | 4,194 | -0.457 | *MACROD2* |  |  |  |  |
|  | 31,183,376 | 31,212,299 | 28,923 | 0.6715 | *LOC149950* |  |  |  |  |
|  | 35,237,305 | 35,276,087 | 38,782 | 0.5124 | *C20orf24* | *SLA2* |  |  |  |
| chr22 | 18,894,543 | 21,454,765 | 2,560,222 | -0.6126 | *DGCR6* | *PRODH* | *DGCR5* | *DGCR9* | *DGCR10* |
|  |  |  |  |  | *DGCR2* | *DGCR11* | *DGCR14* | *TSSK2* | *GSC2* |
|  |  |  |  |  | *SLC25A1* | *CLTCL1* | *HIRA* | *MRPL40* | *C22orf39* |
|  |  |  |  |  | *UFD1L* | *CDC45* | *CLDN5* | *CLDN5* | *LOC150185* |
|  |  |  |  |  | *SEPT5* | *GP1BB* | *TBX1* | *GNB1L* | *C22orf29* |
|  |  |  |  |  | *TXNRD2* | *COMT* | *ARVCF* | *C22orf25* | *MIR185* |
|  |  |  |  |  | *DGCR8* | *MIR1306* | *TRMT2A* | *RANBP1* | *ZDHHC8* |
|  |  |  |  |  | *LOC150197* | *RTN4R* | *MIR1286* | *DGCR6L* | *PI4KAP1* |
|  |  |  |  |  | *RIMBP3* | *ZNF74* | *SCARF2* | *KLHL22* | *MED15* |
|  |  |  |  |  | *POM121L4P* | *TMEM191A* | *PI4KA* | *SERPIND1* | *SNAP29* |
|  |  |  |  |  | *CRKL* | *AIFM3* | *LZTR1* | *THAP7* | *FLJ39582* |
|  |  |  |  |  | *MGC16703* | *P2RX6* | *SLC7A4* | *P2RX6P* |  |
|  | 24,024,415 | 24,028,660 | 4,245 | 0.8936 | *LOC91316* |  |  |  |  |
|  | 24,348,069 | 24,394,680 | 46,611 | 1.1506 | *LOC391322* | *GSTT1* | *GSTTP2* |  |  |
|  | 39,364,491 | 39,369,334 | 4,843 | -1.7084 | *APOBEC3A* |  |  |  |  |
|  | 45,065,451 | 45,137,380 | 71,929 | 0.5317 | *PRR5* | *ARHGAP8* |  |  |  |

**Table S3.** Copy number variations detected by CGH array in schizophrenia patients with 22q11.2 deletions (KO001)

| *Chromosome* | *Start* | *Stop* | *Size* | *Mean of log_2_(ratio)* | *Involved genes* |  |  |  |  |  |
| --- | --- | --- | --- | --- | --- | --- | --- | --- | --- | --- |
| chr1 | 104,093,926 | 104,121,140 | 27,214 | -0.6737 | *RNPC3* |  | *AMY2B* | *LOC648740* |  |  |
|  | 197,546,376 | 197,576,228 | 29,852 | -0.5876 | *DENND1B* |  |  |  |  |  |
| chr2 | 75,744,621 | 75,783,210 | 38,589 | 0.6936 | *FAM176A* |  |  |  |  |  |
|  | 215,224,654 | 215,227,975 | 3,321 | -2.0967 | *SPAG16* |  |  |  |  |  |
| chr3 | 37,464,251 | 37,474,387 | 10,136 | 1.0594 | *C3orf35* |  |  |  |  |  |
|  | 74,481,395 | 74,491,825 | 10,430 | -1.4831 | *CNTN3* |  |  |  |  |  |
| chr4 | 69,341,027 | 69,483,981 | 142,954 | -0.9404 | *TMPRSS11E* |  | *UGT2B17* | *UGT2B15* |  |  |
|  | 87,837,381 | 87,914,417 | 77,036 | -0.4289 | *AFF1* |  |  |  |  |  |
| chr5 | 38,552,840 | 38,557,943 | 5,103 | -1.4836 | *LIFR* |  |  |  |  |  |
|  | 53,388,589 | 53,408,700 | 20,111 | -0.5822 | *ARL15* |  |  |  |  |  |
|  | 82,589,968 | 82,603,102 | 13,134 | -0.4911 | *XRCC4* |  |  |  |  |  |
|  | 131,769,857 | 131,821,036 | 51,179 | 0.5946 | *C5orf56* |  | *IRF1* |  |  |  |
|  | 142,360,493 | 142,404,624 | 44,131 | 0.6887 | *ARHGAP26* |  |  |  |  |  |
|  | 166,961,687 | 166,964,975 | 3,288 | 0.6365 | *ODZ2* |  |  |  |  |  |
| chr6 | 25,391,525 | 25,432,678 | 41,153 | 0.5567 | *LRRC16A* |  |  |  |  |  |
|  | 44,147,857 | 44,277,916 | 130,059 | 0.6585 | *CAPN11* |  | *SLC29A1* | *HSP90AB1* | *SLC35B2* | *NFKBIE* |
|  |  |  |  |  | *TMEM151B* |  | *TCTE1* | *AARS2* |  |  |
| chr7 | 77,843,339 | 77,851,640 | 8,301 | 0.5531 | *MAGI2* |  |  |  |  |  |
|  | 107,425,805 | 107,463,217 | 37,412 | -0.4547 | *SLC26A3* |  |  |  |  |  |
| chr8 | 6,612,659 | 6,618,496 | 5,837 | -0.6948 | *AGPAT5* |  |  |  |  |  |
|  | 18,697,980 | 18,710,315 | 12,335 | 0.7443 | *PSD3* |  |  |  |  |  |
|  | 39,234,811 | 39,388,789 | 153,978 | -0.6268 | *ADAM5P* |  | *ADAM3A* |  |  |  |
|  | 68,074,360 | 68,127,486 | 53,126 | 0.5082 | *CSPP1* |  | *ARFGEF1* |  |  |  |
| chr9 | 130,151,211 | 130,301,946 | 150,735 | 0.5178 | *GARNL3* |  | *SLC2A8* | *ZNF79* | *RPL12* | *SNORA65* |
|  |  |  |  |  | *LRSAM1* |  | *FAM129B* |  |  |  |
| chr11 | 33,106,610 | 33,111,164 | 4,554 | 0.6886 | *CSTF3* |  |  |  |  |  |
|  | 55,339,319 | 55,448,281 | 108,962 | -0.4818 | *OR4C16* |  | *OR4C11* | *OR4P4* | *OR4S2* | *OR4C6* |
|  | 78,062,213 | 78,073,057 | 10,844 | -1.2769 | GAB2 |  |  |  |  |  |
|  | 110,305,002 | 110,349,924 | 44,922 | -0.4506 | FDX1 |  |  |  |  |  |
| chr12 | 72,053,687 | 72,063,982 | 10,295 | 0.5187 | ZFC3H1 |  | *THAP2* |  |  |  |
| chr13 | 108,916,140 | 108,928,456 | 12,316 | -0.7758 | TNFSF13B |  |  |  |  |  |
| chr14 | 20,304,725 | 20,411,895 | 107,170 | 1.3207 | OR4K2 |  | *OR4K5* | *OR4K1* |  |  |
| chr16 | 13,086,807 | 13,122,896 | 36,089 | -0.7222 | SHISA9 |  |  |  |  |  |
|  | 29,984,657 | 30,040,957 | 56,300 | 0.8347 | TAOK2 |  | *HIRIP3* | *INO80E* | *DOC2A* | *C16orf92* |
|  |  |  |  |  | FAM57B |  |  |  |  |  |
|  | 84,176,543 | 84,197,862 | 21,319 | -0.7691 | HSDL1 |  | *LRRC50* |  |  |  |
| chr17 | 12,552,062 | 12,571,036 | 18,974 | 0.7 | MYOCD |  |  |  |  |  |
|  | 16,777,765 | 16,859,788 | 82,023 | 0.631 | TNFRSF13B |  |  |  |  |  |
|  | 19,568,297 | 19,581,760 | 13,463 | -0.497 | ALDH3A2 |  | *SLC47A2* |  |  |  |
|  | 27,123,801 | 27,129,556 | 5,755 | -0.8177 | C17orf63 |  |  |  |  |  |
|  | 38,556,846 | 38,577,955 | 21,109 | -0.5108 | TOP2A |  |  |  |  |  |
|  | 39,186,628 | 39,191,490 | 4,862 | -0.9837 | KRTAP1-3 |  |  |  |  |  |
|  | 58,301,541 | 58,321,122 | 19,581 | -0.6542 | USP32 |  | *SCARNA20* |  |  |  |
|  | 61,271,800 | 61,280,788 | 8,988 | -0.9743 | TANC2 |  |  |  |  |  |
|  | 63,153,372 | 63,170,411 | 17,039 | 1.0489 | RGS9 |  |  |  |  |  |
|  | 65,065,579 | 65,157,493 | 91,914 | -0.4192 | HELZ |  |  |  |  |  |
|  | 71,774,635 | 71,784,757 | 10,122 | 0.6175 | C17orf54 |  |  |  |  |  |
| chr18 | 24,106,394 | 24,113,228 | 6,834 | 0.8063 | KCTD1 |  |  |  |  |  |
| chr20 | 1,562,887 | 1,576,390 | 13,503 | -1.4459 | SIRPB1 |  |  |  |  |  |
|  | 21,487,198 | 21,498,705 | 11,507 | 0.7112 | NKX2-2 |  |  |  |  |  |
| chr21 | 27,106,500 | 27,112,411 | 5,911 | 0.5338 | ATP5J |  | *GABPA* |  |  |  |
| chr22 | 18,894,551 | 21,441,974 | 2,547,423 | -0.4923 | DGCR6 |  | *PRODH* | *DGCR5* | *DGCR9* | *DGCR10* |
|  |  |  |  |  | DGCR2 |  | *DGCR11* | *DGCR14* | *TSSK2* | *TSSK2* |
|  |  |  |  |  | SLC25A1 |  | *CLTCL1* | *HIRA* | *MRPL40* | *C22orf39* |
|  |  |  |  |  | UFD1L |  | *CDC45* | *CLDN5* | *CLDN5* | *LOC150185* |
|  |  |  |  |  | SEPT5 |  | *GP1BB* | *TBX1* | *GNB1L* | *C22orf29* |
|  |  |  |  |  | TXNRD2 |  | *COMT* | *ARVCF* | *C22orf25* | *MIR185* |
|  |  |  |  |  | DGCR8 |  | *MIR1306* | *TRMT2A* | *RANBP1* | *ZDHHC8* |
|  |  |  |  |  | *LOC150197* |  | *RTN4R* | *MIR1286* | *DGCR6L* | *PI4KAP1* |
|  |  |  |  |  | *RIMBP3* |  | *ZNF74* | *SCARF2* | *KLHL22* | *MED15* |
|  |  |  |  |  | *POM121L4P* |  | *TMEM191A* | *PI4KA* | *SERPIND1* | *SERPIND1* |
|  |  |  |  |  | *CRKL* |  | *AIFM3* | *LZTR1* | *THAP7* | *FLJ39582* |
|  |  |  |  |  | *MGC16703* |  | *P2RX6* | *SLC7A4* | *P2RX6P* | *LOC400891* |

**Table S4.** Expression of genes located at the 22q11.2 deletion region in patient-derived neurospheres

| *Gene name* | P*-value* | *Fold Change* | *Regulation* | *Chr: Start Position (bp)* |
| --- | --- | --- | --- | --- |
| *DGCR11* | 0.031 | 0.776 | down | 22:19033652 |
| *DGCR14* | 0.002 | 0.540 | down | 22:19121450 |
| *SLC25A1* | 0.004 | 0.576 | down | 22:19163119 |
| *HIRA* | 0.004 | 0.516 | down | 22:19318223 |
| *CDC45* | 0.017 | 0.513 | down | 22:19467467 |
| *CLDN5* | 0.013 | 0.758 | down | 22:19510549 |
| *GNB1L* | 0.022 | 0.850 | down | 22:19775945 |
| *C22orf29* | 0.010 | 0.545 | down | 22:19833668 |
| *DGCR8* | 0.005 | 0.640 | down | 22:20098441 |
| *TRMT2A* | 0.027 | 0.852 | down | 22:20099826 |
| *RANBP1* | 0.024 | 0.677 | down | 22:20104919 |
| *ZDHHC8* | 0.037 | 0.731 | down | 22:20127007 |
| *RTN4R* | 0.008 | 0.624 | down | 22:20228939 |
| *PI4KA* | 0.030 | 0.674 | down | 22:20383523 |
| *ZNF74* | 0.035 | 0.660 | down | 22:20762027 |
| *KLHL22* | 0.027 | 0.753 | down | 22:20795816 |
| *MED15* | 0.048 | 0.751 | down | 22:20936187 |
| *PI4KA* | 0.012 | 0.661 | down | 22:21061990 |
| *SNAP29* | 0.022 | 0.669 | down | 22:21213324 |
| *CRKL* | 0.036 | 0.619 | down | 22:21305017 |
| *LZTR1* | 0.017 | 0.796 | down | 22:21336664 |
| *THAP7* | 0.008 | 0.709 | down | 22:21354060 |

Abbreviation: Chr, chromosome

**Table S5.** Significantly enriched GO terms in patient-derived neurospheres

| *GO ID* | *Term* | *P value* | *Number of input genes* |
| --- | --- | --- | --- |
| GO:0048519 | Negative regulation of biological process | 1.7E-07 | 105 |
| GO:0045595 | Regulation of cell differentiation | 4.5E-07 | 49 |
| GO:0045596 | Negative regulation of cell differentiation | 3.6E-05 | 28 |
| GO:0051093 | Negative regulation of developmental process | 3.1E-04 | 30 |
| GO:0051093 | Negative regulation of developmental process | 3.1E-04 | 30 |
| GO:0012501 | Programmed cell death | 3.2E-03 | 38 |
| GO:0045202 | Synapse | 4.3E-03 | 24 |
| GO:0043005 | Neuron projection | 4.3E-03 | 29 |
| GO:0097458 | Neuron part | 1.7E-02 | 31 |
| GO:0007399 | Nervous system development | 1.8E-02 | 52 |
| GO:0031053 | Primary miRNA processing | 3.2E-02 | 5 |
| GO:0030855 | Epithelial cell differentiation | 4.5E-02 | 17 |
| GO:0048731 | System development | 4.5E-02 | 81 |

GO, Gene Ontology

**Table S6.** Top 15 up-regulated or down-regulated pathways in patient-derived neurospheres

|  | *Name of the event* | *Hit genes in each event* | *Total number of genes in each event* | P-*value* |
| --- | --- | --- | --- | --- |
| Up-regulated pathway |  |  |  |  |
| 1 | MAPK targets/ Nuclear events mediated by MAP kinases | 6 | 30 | 2.54E-05 |
| 2 | Neurotransmitter receptor binding and downstream transmission | 12 | 143 | 3.06E-05 |
| 3 | Transmission across Chemical Synapses | 14 | 196 | 4.04E-05 |
| 4 | NGF signaling via TRKA from the plasma membrane | 14 | 207 | 7.36E-05 |
| 5 | Activation of NMDA receptor upon glutamate binding and postsynaptic events | 6 | 39 | 1.20E-05 |
| 6 | Signaling by NGF | 16 | 290 | 2.47E-04 |
| 7 | Neuronal System | 16 | 292 | 2.67E-04 |
| 8 | Unblocking of NMDA receptor, glutamate binding and activation | 4 | 17 | 3.19E-04 |
| 9 | Trafficking of AMPA receptors | 5 | 31 | 3.61E-04 |
| 10 | Glutamate Binding, Activation of AMPA Receptors and Synaptic Plasticity | 5 | 31 | 3.61E-04 |
| 11 | Signaling by SCF-KIT | 10 | 142 | 5.97E-04 |
| 12 | ERK/MAPK targets | 4 | 21 | 7.53E-04 |
| 13 | MAP kinase activation in TLR cascade | 6 | 55 | 8.17E-04 |
| 14 | Signaling by EGFR | 11 | 179 | 1.00E-03 |
| 15 | Signaling by EGFR in Cancer | 11 | 181 | 1.10E-03 |
| Down-regulated pathway |  |  |  |  |
| 1 | Cell Cycle | 61 | 508 | 4.14E-23 |
| 2 | Cell Cycle, Mitotic | 52 | 411 | 1.45E-20 |
| 3 | G2/M Checkpoints | 18 | 48 | 3.70E-16 |
| 4 | Mitotic M-M/G1 phases | 36 | 266 | 3.62E-15 |
| 5 | DNA strand elongation | 14 | 31 | 3.54E-14 |
| 6 | Activation of ATR in response to replication stress | 15 | 41 | 1.82E-13 |
| 7 | Activation of the pre-replicative complex | 13 | 32 | 1.61E-12 |
| 8 | Synthesis of DNA | 20 | 95 | 1.94E-12 |
| 9 | Cell Cycle Checkpoints | 23 | 131 | 2.22E-12 |
| 10 | S Phase | 22 | 122 | 3.94E-12 |
| 11 | DNA Replication | 20 | 102 | 7.89E-12 |
| 12 | Unwinding of DNA | 7 | 11 | 4.87E-09 |
| 13 | G1/S Transition | 17 | 113 | 2.20E-08 |
| 14 | Telomere C-strand (Lagging Strand) Synthesis | 8 | 22 | 1.03E-07 |
| 15 | Gap-filling DNA repair synthesis and ligation in GG-NER | 7 | 16 | 1.49E-07 |

**Table S7.** Prediction of miRNAs that target *MAPK14* by TargetScan release 7.0^a^

| *miRNA* | *Seed match* | *Context++ score* | *Context++ score percentile* | *Weighted context++ score* | *Conserved branch length* | *Probability of conserved targeting* |
| --- | --- | --- | --- | --- | --- | --- |
| *hsa-miR-216a-3p* | 8mer | -0.35 | 98 | -0.35 | 6.678 | 0.9 |
| *hsa-miR-128-3p* | 8mer | -0.31 | 98 | -0.31 | 6.678 | 0.9 |
| *hsa-miR-3681-3p* | 8mer | -0.34 | 98 | -0.34 | 6.678 | 0.9 |
| *hsa-miR-27b-3p* | 7mer-m8 | -0.1 | 81 | -0.1 | 6.678 | 0.76 |
| *hsa-miR-27a-3p* | 7mer-m8 | -0.1 | 81 | -0.1 | 6.678 | 0.76 |
| *hsa-miR-185-5p*^b^ | 7mer-1A | -0.17 | 85 | -0.17 | 4.068 | N/A |
| *hsa-miR-4644* | 7mer-1A | -0.17 | 85 | -0.17 | 4.068 | N/A |
| *hsa-miR-4306* | 7mer-1A | -0.17 | 85 | -0.17 | 4.068 | N/A |
| *hsa-miR-582-5p* | 8mer | -0.23 | 96 | -0.22 | 2.363 | N/A |
| *hsa-miR-101-3p.2* | 7mer-1A | -0.13 | 90 | -0.13 | 3.645 | 0.27 |
| *hsa-miR-124-3p.1* | 8mer | -0.5 | 99 | -0.48 | 5.98 | 0.97 |
| *hsa-miR-124-3p.2* | 8mer | -0.36 | 94 | -0.35 | 5.301 | 0.85 |
| *hsa-miR-506-3p* | 8mer | -0.3 | 94 | -0.29 | 5.301 | 0.85 |
| *hsa-miR-24-3p* | 7mer-m8 | -0.27 | 95 | -0.26 | 3.663 | 0.49 |
| *hsa-miR-505-3p.2* | 8mer | -0.11 | 93 | -0.11 | 2.234 | N/A |
| *hsa-miR-6835-3p* | 7mer-m8 | -0.06 | 85 | -0.05 | 2.826 | N/A |
| *hsa-miR-22-3p* | 7mer-m8 | -0.43 | 98 | -0.41 | 3.801 | 0.54 |
| *hsa-miR-340-5p* | 8mer | -0.09 | 96 | -0.09 | 1.836 | N/A |
| *hsa-miR-125a-5p* | 7mer-m8 | -0.26 | 93 | -0.25 | 3.416 | 0.41 |
| *hsa-miR-125b-5p* | 7mer-m8 | -0.22 | 90 | -0.21 | 3.416 | 0.41 |
| *hsa-miR-4319* | 7mer-m8 | -0.18 | 86 | -0.16 | 3.416 | 0.41 |
| *hsa-miR-19b-3p* | 8mer | -0.18 | 81 | -0.17 | 2.817 | 0.3 |
| *hsa-miR-19a-3p* | 8mer | -0.18 | 81 | -0.17 | 2.817 | 0.3 |
| *hsa-miR-489-3p* | 7mer-m8 | -0.22 | 97 | -0.2 | 5.245 | 0.14 |

Twenty-four miRNAs that have conserved sites are shown.

^a^http://www.targetscan.org/

^b^*has-miR-185-5p* is reported to be significantly down-regulated similar to our study^1^.

**Supplementary Figures**


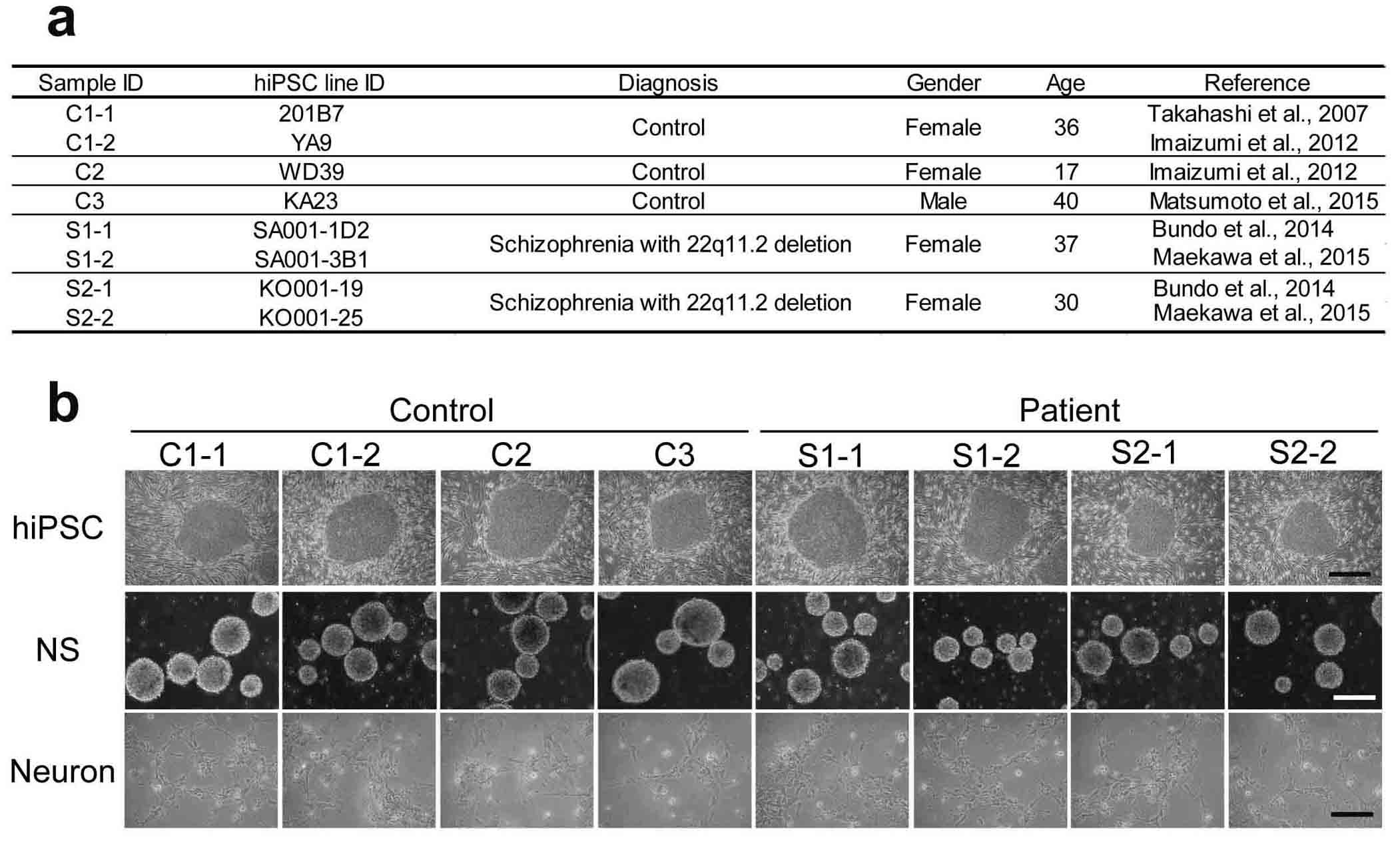


**Figure S1.** Establishment of hiPSCs and hiPSC-derived cells from controls and schizophrenia patients with a 22q11.2 deletion. (**a**) Summary of the hiPSC lines. (**b**) Control hiPSC lines (C1-1, C1-2, C2 and C3) and patient-derived hiPSC lines (S1-1, S1-2, S2-1 and S2-2) could be differentiated into neurons via neurosphere formation. Scale bars: hiPSC and neuron, 400 μm; neurospheres, 150 μm.

**
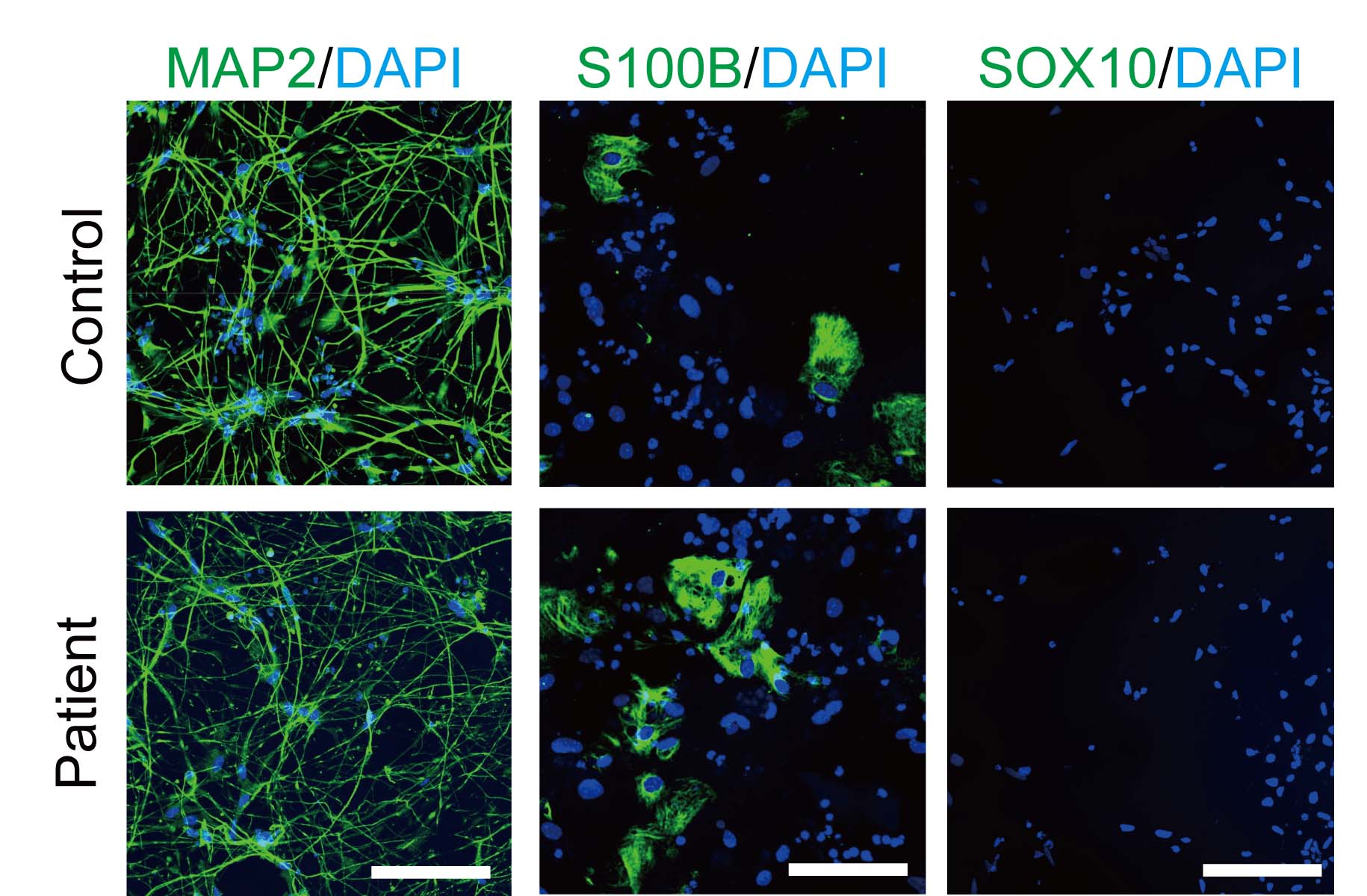
**

**Figure S2.** Representative images of differentiated cells from neurospheres. Neural cells derived from neurospheres expressed MAP2 (a neuronal marker) and S100B (an astrocyte marker), but not SOX10 (an oligodendrocyte marker), in patient (lower panel) and control (upper panel)-derived samples. Scale bars: 50 µm.


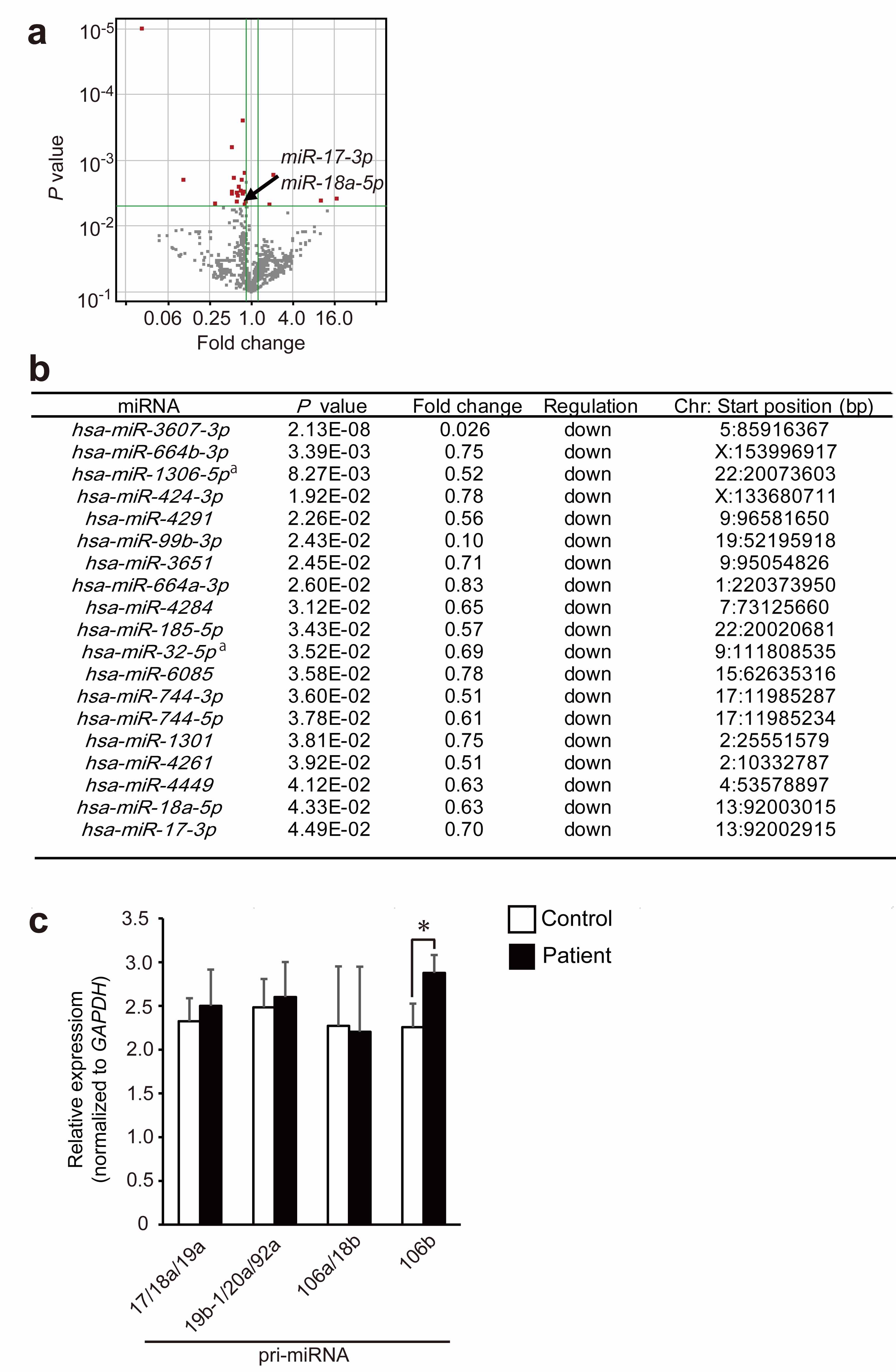


**Figure S3.** miRNA biogenesis alterations in patient-derived neurospheres. (**a**) Changes in miRNA expression in the neurospheres derived from patients and control. Volcano plot of the *P* values and the corresponding relative expression of each miRNA. The *P* values for differences in gene expression were calculated using the *t-*test and plotted as log (*P*). Red dots, absolute 1.2-fold cutoff (*P* < 0.05). (**b**) Summary of miRNA whose expression changed significantly in neurospheres. Upper a (^a^) show the miRNAs that are reported to be significantly changed with the same direction as in our study^1^. (**c**) Quantitative RT-PCR analysis of pri-miRNAs in neurosphere. *GAPDH* mRNA was used as an internal control (*n* = 4). Error bars show mean ± SEM (**P* < 0.05; two-tailed *t*-test).


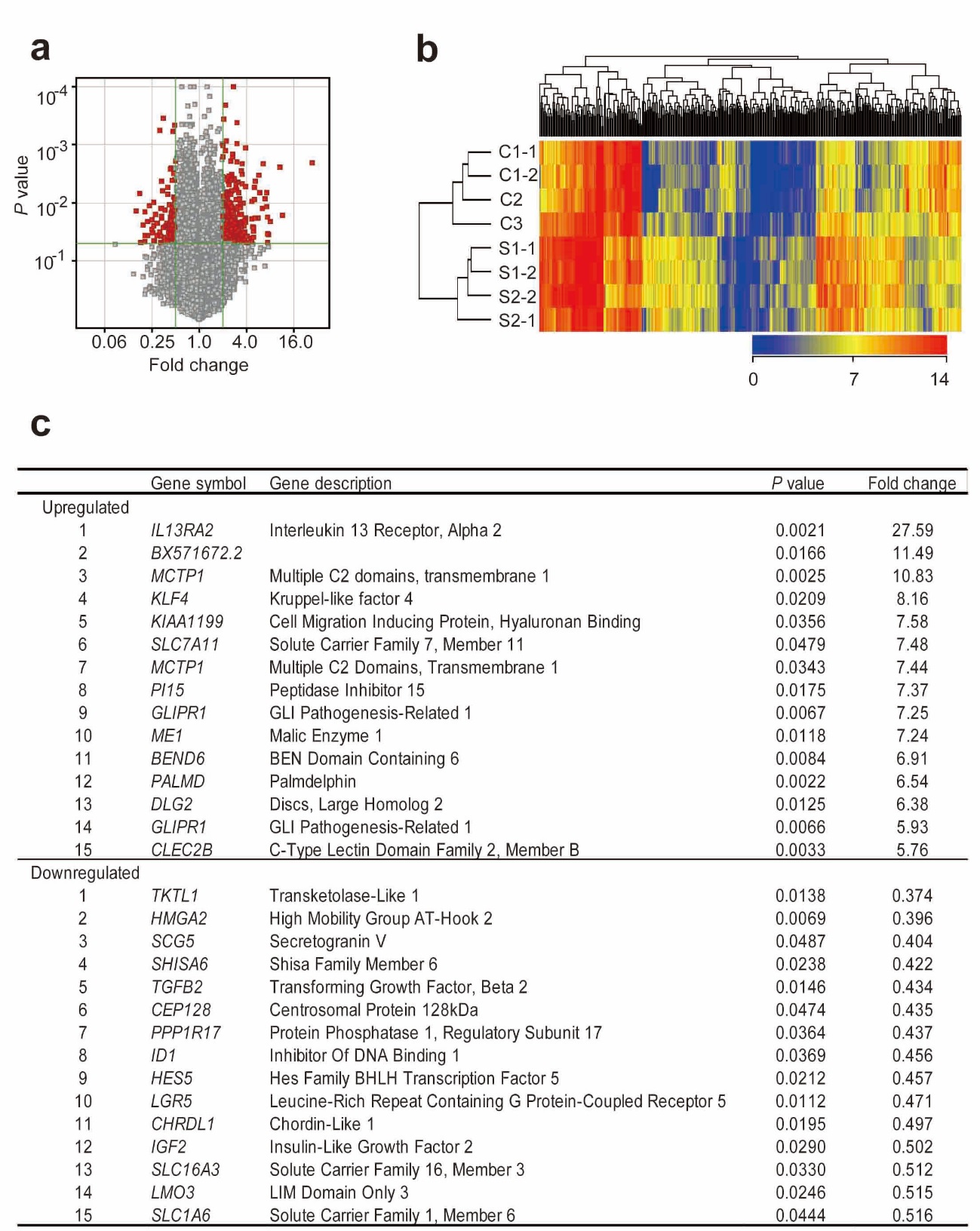


**Figure S4.** Changes in the transcriptome of patient-derived neurospheres. (**a**) Changes in gene expression in patient and control derived neurospheres. Volcano plot of the *P* values and the corresponding relative expression of each miRNA. The *P*-values for differences in gene expression were calculated using the *t-*test and plotted as log (*P*). Red dots, absolute 2-fold cutoff (*P* < 0.05). (**b**) Heat map showing differential expression of 386 unique genes (263 upregulated and 123 downregulated) between patient and control neurospheres. (**c**) Summary of the top 15 genes that were significantly upregulated or downregulated. These genes ranked by fold-change.

**
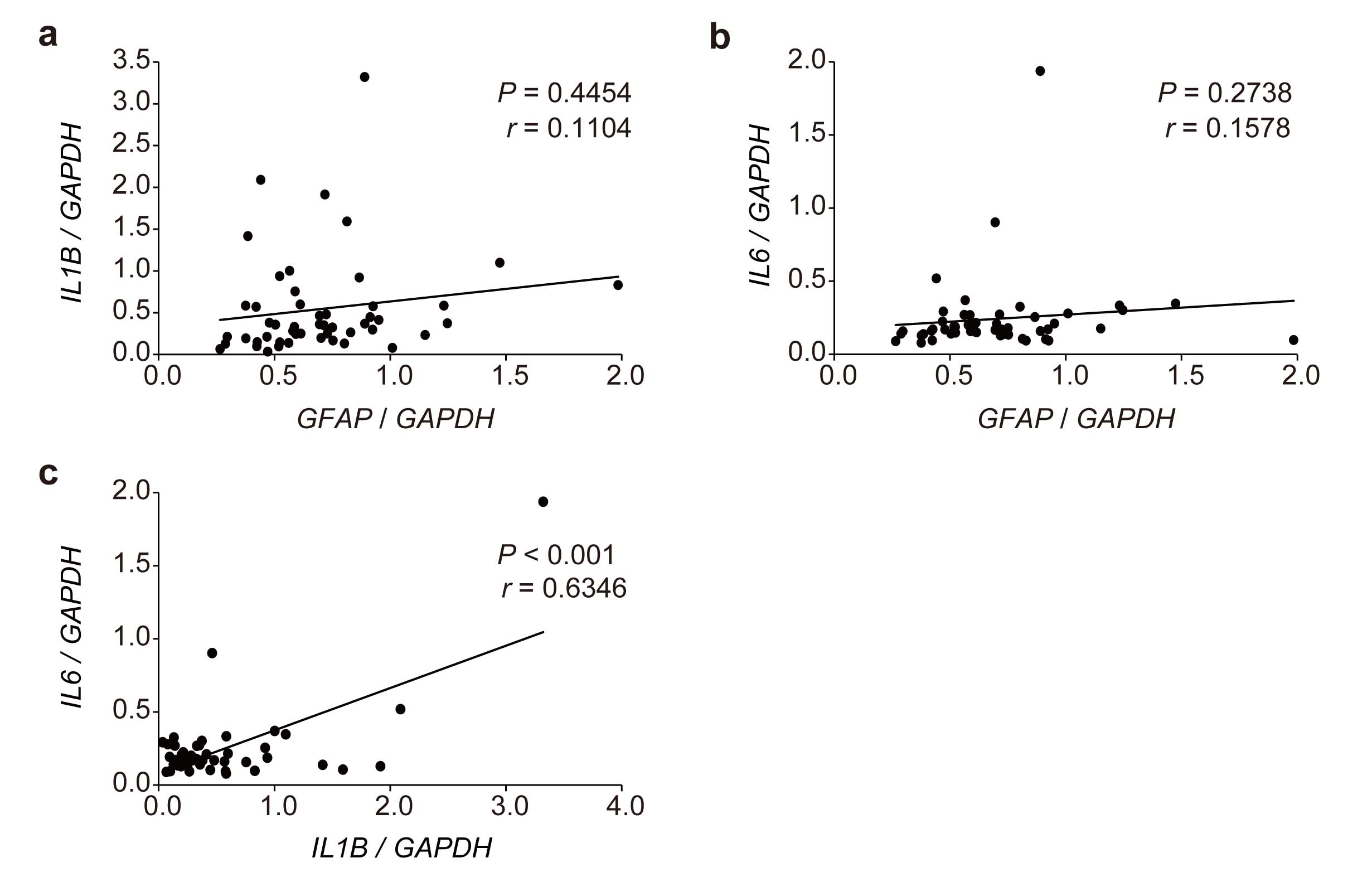
**

**Figure S5.**  Correlation analyses (Pearson's method) of *GFAP*, *IL1B* and *IL6* expression in schizophrenia brains. (**a**) Correlation between *GFAP* and *IL1B*. (**b**) Correlation between *GFAP* and *IL6.* (**c**) Correlation between *IL1B* and *IL6*.

**
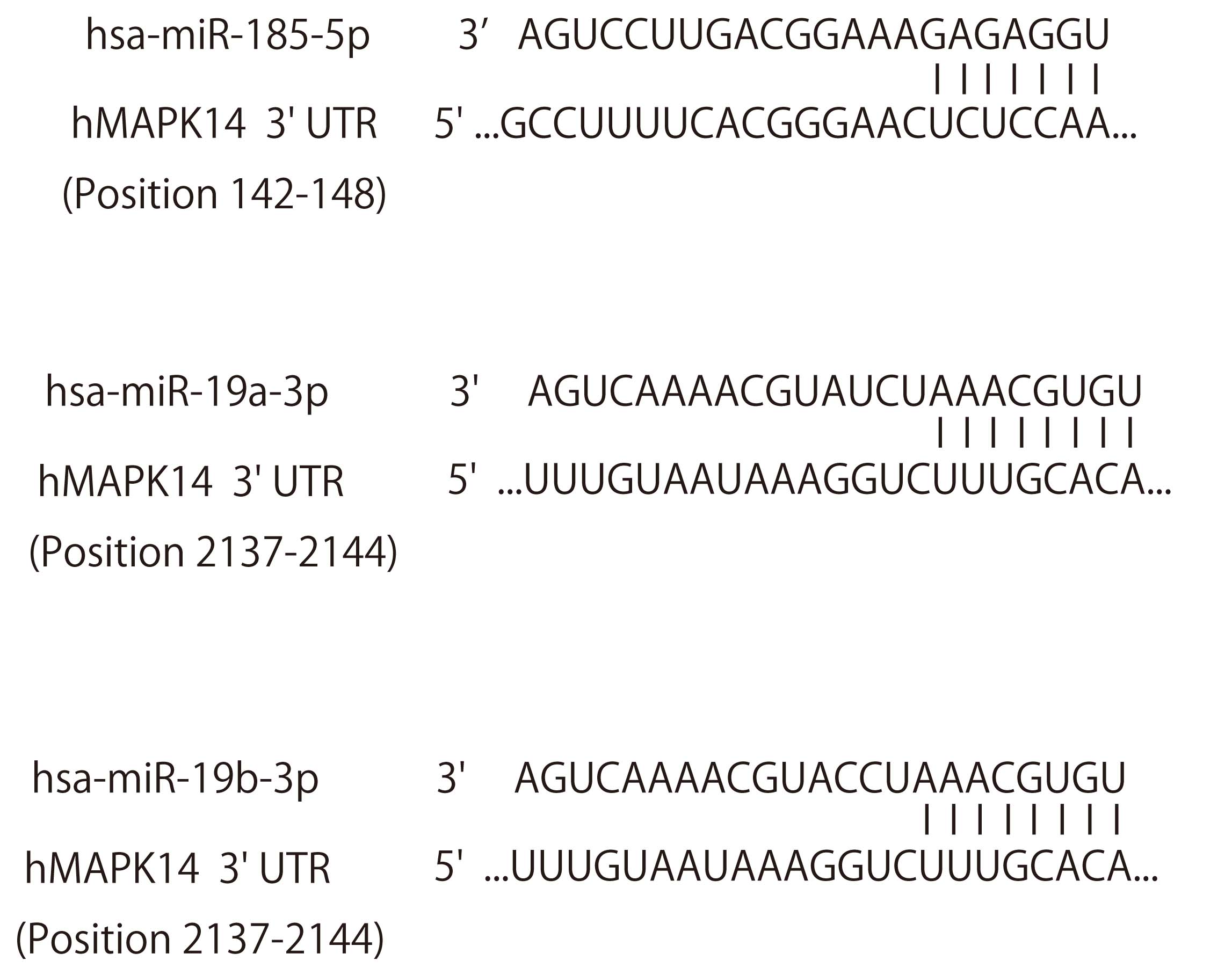
**

**Figure S6.**  Schematic diagrams showing binding between miR-185 and miR-19a/b, and the 3’UTR of human *MAPK14* mRNA.

**REFERENCES**

1. Zhao D, Lin M, Chen J, Pedrosa E, Hrabovsky A, Fourcade HM*, et al*. MicroRNA Profiling of Neurons Generated Using Induced Pluripotent Stem Cells Derived from Patients with Schizophrenia and Schizoaffective Disorder, and 22q11.2 Del. *PLoS One* 2015; **10:** e0132387.
